# The Apoptotic Effect of 1’*S*-1’-Acetoxychavicol Acetate from *Alpinia Conchigera* on Human Cancer Cells

**DOI:** 10.3390/molecules15118048

**Published:** 2010-11-09

**Authors:** Khalijah Awang, Mohamad Nurul Azmi, Lionel In Lian Aun, Ahmad Nazif Aziz, Halijah Ibrahim, Noor Hasima Nagoor

**Affiliations:** 1 Centre for Natural Product Research and Drug Discovery (CENAR), Department of Chemistry, Faculty of Science, University Malaya, 50603 Kuala Lumpur, Malaysia; E-Mail: libra_mine85@yahoo.co.uk (M.N.A.); 2 Institute of Biological Sciences (Genetics & Molecular Biology), Faculty of Science, University Malaya, 50603, Kuala Lumpur, Malaysia; E-Mails: lionelin_81@yahoo.com (L.I.L.A.); hasima@um.edu.my (N.H.N.); 3 Department of Chemical Science, Faculty of Science and Technology, University Malaysia Terengganu, Mengabang Telipot, 2103, Kuala Terengganu, Malaysia; E-Mail: nazif@umt.edu.my (A.N.A.); 4 Institute of Biological Sciences (Ecology and Biodiversity), Faculty of Science, University Malaya, 50603, Kuala Lumpur, Malaysia; E-Mail: ihalijah@um.edu.my (H.I.)

**Keywords:** 1’-(*S*)-1’-acetoxychavicol acetate, *Alpinia conchigera*, apoptosis, cytotoxicity, cell cycle arrest

## Abstract

1’-(*S*)-1’-Acetoxychavicol acetate (ACA) isolated from the Malaysian ethno-medicinal plant *Alpinia conchigera* Griff. was investigated for its potential as an anticancer drug. In this communication, we describe the cytotoxic and apoptotic properties of ACA on five human tumour cell lines. Data from MTT cell viability assays indicated that ACA induced both time- and dose-dependent cytotoxicity on all tumour cell lines tested and had no adverse cytotoxic effects on normal cells. Total mortality of the entire tumour cell population was achieved within 30 hrs when treated with ACA at 40.0 µM concentration. Flow cytometric analysis for annexin-V and PI dual staining demonstrated that cell death occurred via apoptosis, followed by secondary necrosis. The apoptotic effects of ACA were confirmed via the DNA fragmentation assay, in which consistent laddering of genomic DNA was observed for all tumour cell lines after a 24 hrs post-treatment period at the IC_50_ concentration of ACA. A cell cycle analysis using PI staining also demonstrated that ACA induced cell cycle arrest at the G_0_/G_1_ phase, corresponding to oral tumour cell lines. In conclusion, ACA exhibits enormous potential for future development as a chemotherapeutic drug against various malignancies.

## 1. Introduction

Medicinal herbs have always been used as traditional primary health care agents, especially in Asian countries, and over the last 20 years, there have been rapid changes in the popularity of the use of natural systems to maintain health and for alternative therapy [1,2]. However, scientific studies on the use of most traditional medicinal plants to ensure their efficacy and non-toxicity have not been carried out.

The plant *Alpinia conchigera* Griff. (Family: Zingiberaceae; Section: Zingiberacea; Sub-section: Strobidia) is known locally as *lengkuas ranting*, *lengkuas kecil*, *lengkuas padang*, *lengkuas geting* or *chengkenam* [3,4,5]. It is a herbaceous perennial, 2-5 ft. tall, found in Eastern Bengal and southward towards Peninsular Malaysia and Sumatera (Sumatra) [6,7]. It is used as a condiment in the northern state of Peninsular Malaysia and occasionally in traditional medicine on the east coast to treat fungal infections [8]. In Thailand, the rhizomes are used in traditional Thai medicine to relieve gastro-intestinal disorders and in the preparation of Thai food dishes [9]. 

**Figure 1:**
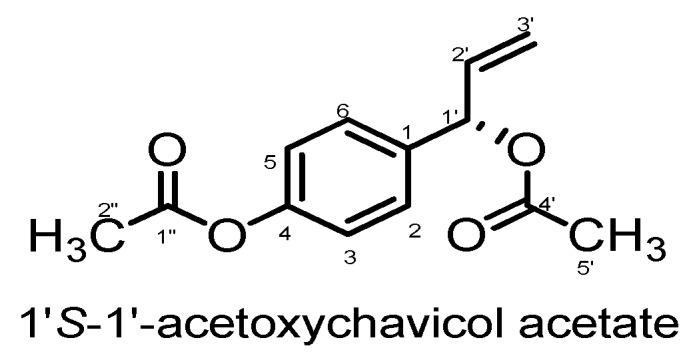
Chemical structure of 1’*S*-1’-acetoxychavicol acetate.

1’-(*S*)-1’-Acetoxychavicol acetate (ACA, Figure 1), which can be isolated from *Alpinia conchigera,* has been the subject of various biological activity studies. One of the early studies with this compound showed its *in vitro* inhibitory effects on tumour-promoted-induced Epstein-Barr virus activation [10]. Subsequent studies demonstrated that ACA inhibits Ehrlich ascites tumour cells, skin tumour promotion and adenocarcinoma formation [11,12,13,14]. More recently, ACA was shown to inhibit the cellular growth of the hematological malignant cells *in vitro* and *in vivo* by the induction of apoptosis via mitochondrial- and Fas-mediated dual mechanism in myeloid leukemic cells [15]. Because of our interest in the antitumourigenic properties of traditional herbs, our goal was to investigate the potential of ACA to induce apoptosis in various human tumour cells: breast adenocarcinoma (MCF-7), oral squamous carcinoma (HSC-2 and HSC-4), hepatocyte carcinoma (HepG2) and epidermoid cervical carcinoma (CaSki). This communication therefore describes the cytotoxic and apoptotic properties of 1’*S*-1’-acetoxychavicol acetate on various human malignancies *in vitro* to better understand its potential therapeutic value.

## 2. Results and Discussions

### 2.1. LC-MS Analysis

**Figure 2:**
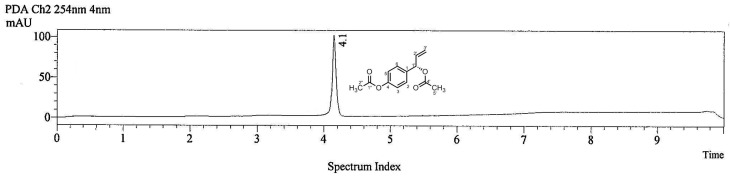
Chromatogram of 1’*S*-1’-acetoxychavicol acetate at 254 nm.

1’-(*S*)-1’-Acetoxychavicol acetate (ACA) was injected into an LC-MS system to give a chromatogram (Figure 2) recorded at 254 nm, in which ACA gave a strong peak at *t*_R_ = 4.1 min.

### 2.2. ACA Induced Dose- and Time-Dependent Cytotoxic in Tumour Cells

**Figure 3:**
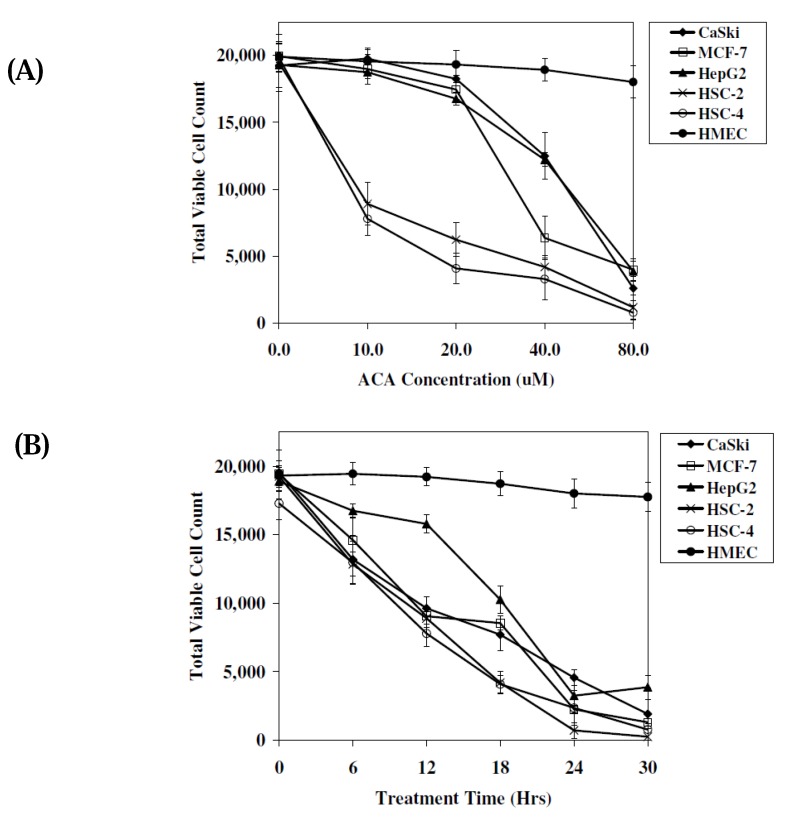
The cytotoxic effects of ACA on tumour cell lines assessed using the MTT cell viability assay. (**A**) Comparison of total viable cell count between various tumour cell lines after treatment with ACA at different concentrations (0–80.0 μM) at 12 hrs post-treatment time. (**B**) Comparison of total viable cell count between various tumor cell lines after treatment with 40.0 μM ACA for 30 hrs. All experiments were plotted as mean values (n = 3).

To determine if cytotoxicity was dose- and time-dependant, the MTT cell viability assay which measures the mitochondrial activity in viable cells was conducted. Results indicated that cells treated with ACA induced cytotoxicity in a dose dependant manner. Highest levels of cytotoxicity were observed in oral squamous carcinoma cells (HSC-4 and HSC-2), with IC_50_ values of 8.0 μM and 9.0 μM, respectively, at 12 hrs post-treatment time (Figure 3A). At 80.0 μM ACA concentration, more than 80% of tumour cells assayed died after 12 hrs of exposure to ACA (Figure 3A). MTT assay data in Figure 3B demonstrated that the cytotoxic effects of ACA on tumour cell lines were also time dependant. At 40.0 μM, all cell lines were killed after a 30 hrs incubation period and the viability of all cell lines were reduced to 40%–50% after 12 hrs of ACA treatment, with the exception of HepG2 cells (Figure 3B). Reductions in cell viability was once again found to be greatest in HSC-4 and HSC-2 cells, indicating that ACA induced cell death most efficiently in oral squamous carcinoma type cells (Figure 3B). The 12 hrs IC_50_ values of ACA in all cancer cell lines with the exception of HSC-2 and HSC-4 oral cancer cell lines, were considerably high, ranging from 34.0 μM to 48.0 μM in MCF-7, HepG2 and CaSki cell lines. This suggested the primary application of ACA against oral carcinomas, consistent with reports indicating the traditional consumption of *Alpinia conchigera* rhizomes orally. Viability of cells treated with DMSO without ACA were insignificantly affected (<1.0%; data not shown), indicating that cytotoxicity was induced by ACA and not the DMSO solvent, which has been reported to be cytotoxic at high concentrations [16]. Figure 3A and 3B also indicated that no adverse cytotoxic effects were observed when treated with HMEC normal human cells with viability levels maintained above 90.0%. Based on initial MTT assay results, the concentration of ACA in all subsequent experiments were set at the IC_50 _value of respective cell lines with a 12 hrs incubation time (Table 1). Both time and dose dependant assays supported the need to further investigate the apoptotic effects of ACA in oral cancer cells.

**Table 1:** IC_50_ values of ACA in various human cancer cell lines at different incubation periods.

Human Cancer Cell Lines	IC_50_ value (µM)	Incubation Time (hours)
Breast adenocarcinoma (MCF-7)	34.0*	12*
	30.0	24
Oral squamous carcinoma (HSC-2)	9.0*	12*
	5.0	24
Oral squamous carcinoma (HSC-4)	8.0*	12*
	5.5	24
Hepatocyte carcinoma (HepG2)	48.0*	12*
	18.0	24
Epidermoid cervical carcinoma (CaSki)	48.0*	12*
	17.0	24

*Denotes treatment parameters used in flow cytometry and DNA fragmentation assays.

### 2.3. ACA Induced-Potential Cell Cycle Arrest at the G_0_/G_1_ Phase

**Figure 4:**
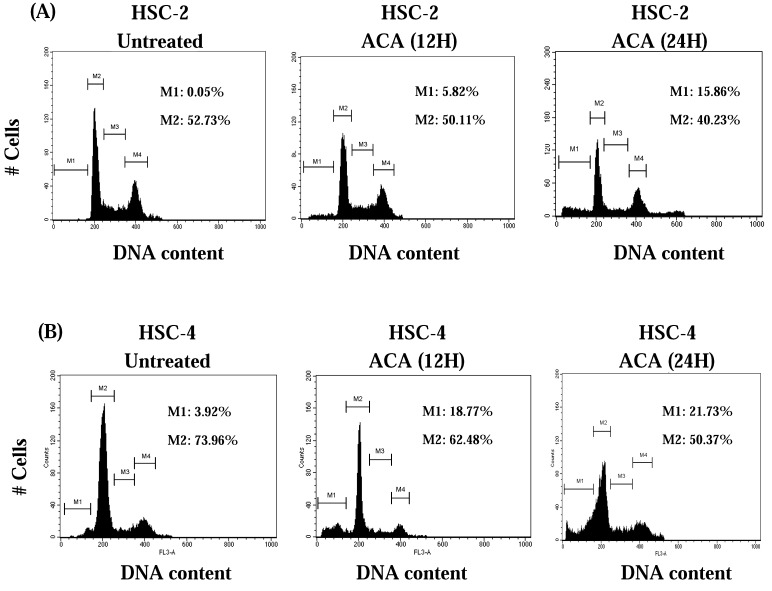
Cell cycle distribution of cancer cell lines using flow cytometry after staining with PI. (A) HSC-2 cells treated with ACA; (B) HSC-4 cells treated with ACA. M1: Sub-G_1_ phase; M2:G_0_/G_1_ phase; M3:S phase; M4:G_2_/M phase. All experiments are a representative of 10,000 cells and the percentage of cells in all cell cycle phases are indicated.

As cytotoxicity was best demonstrated on oral cancer cells, we next compared the cell cycle distribution patterns before and after ACA treatment. Both attached and floating oral cancer cells were stained with propidium iodide and analyzed by flow cytometry. Cultivation with ACA significantly decreased the population of HSC-2 and HSC-4 cells in the G_0_/G_1_ phase, indicating a potential arrest during the pre-DNA replication G_0_/G_1_ checkpoint (Figure 4A and Figure 4B). Both tumour cell lines also demonstrated an indication of apoptosis by the appearance of a hypodiploid DNA peak within the sub-G_1_ phase 24 hrs after ACA exposure (Figure 4A and Figure 4B).

### 2.4. ACA Induced Cell Death via Apoptosis

**Figure 5:**
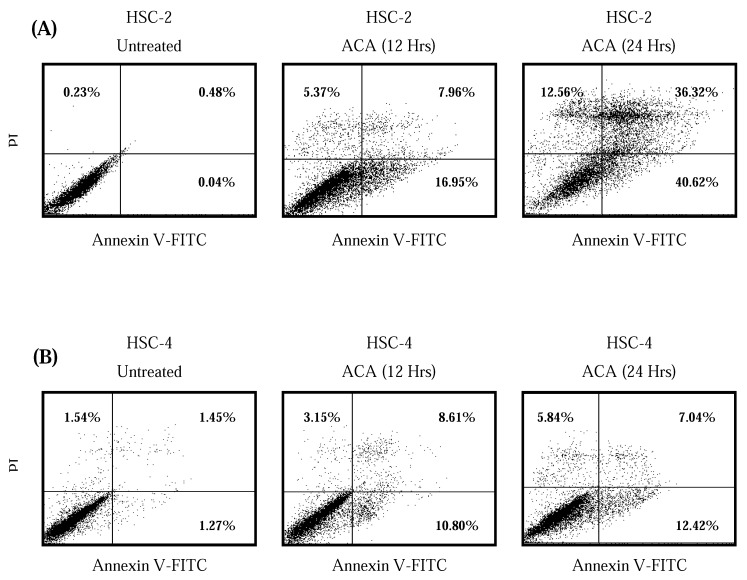
Detection of apoptosis using flow cytometry after annexin V-FITC/ propidium iodide (PI) staining indicating that ACA potentiates apoptosis-mediated cell death after 12 hrs and 24 hrs of exposure. (A) HSC-2 cells treated with ACA; (B) HSC-4 cells treated with ACA. Viable cells are in the lower left quadrant, early apoptotic cells are in the lower right quadrant, late apoptotic cells are in the upper right quadrant and non-viable necrotic cells are in the upper left quadrant. Dot plots are a representative of 10,000 cells from a single replicate.

**Figure 6:**
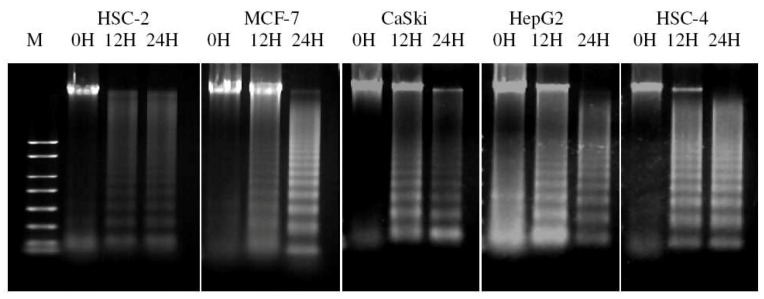
Confirmation of apoptosis using the DNA fragmentation assay. Cells were both untreated and treated with ACA for 12 hrs and 24 hrs to observe its effects on DNA laddering (M: 100 bp DNA Size Marker).

In order to investigate if tumour cells were dying through apoptosis as opposed to primary necrosis, a double fluorescence staining of annexin V-FITC conjugate and propidium iodide was performed on tumour cells before and after IC_50 _ACA treatment, and analyzed using a flow cytometer. After exposure of all five tumour cell lines to ACA for 12 hrs and 24 hrs, the population of cells indicated a shift from viable cells to early stage and late stage apoptosis, followed by secondary necrosis. HSC-2 cells displayed a higher percentage of apoptotic cells with 76.94% compared to HSC-4 cells with 19.46% (Figure 5A and Figure 5B). Agarose gel electrophoresis of DNA extracted from cancer cells treated with ACA demonstrated partial and complete fragmentation of genomic DNA after 12 hrs and 24 hrs, respectively (Figure 6). This confirmed the induction of apoptosis, in which the consistent laddering of genomic DNA represents one of the major hallmarks of apoptosis.

Various plants species have been utilized as medicines for thousands of years, and traditional medicine has been a fertile source of novel lead molecules for modern drug discovery. In this study, MTT and flow cytometric assays were conducted with four main objectives: firstly, to determine whether the cytotoxic activity of ACA, a natural compound isolated from the Malaysian ethno-medicinal plant *Alpinia conchigera* Griff., was dose or time dependant; secondly, to investigate the cytotoxic effects of 1’-(*S*)-1’-acetoxychavicol acetate (ACA) on normal cells; thirdly, to find out whether cell death was induced through apoptosis, and fourthly, to determine the IC_50_ values of ACA in each cancer cell type, which is crucial in subsequent downstream assays. 

Cells undergoing apoptosis can be observed via multiple hallmarks such as the appearance of a sub-diploid DNA peak during PI-based cell cycle analysis, the staining of exposed phosphatidylserine on the cell surface which is demonstrated during annexin V-FITC/PI dual staining flow cytometry, and the consistent laddering of genomic DNA [17,18]. The fact that ACA induced cytotoxicity in a dose and time dependant manner was both important and convenient, as this allowed the manipulation and use of lower drug dosages with longer exposure periods, which can result in fewer side effects on non-cancerous tissue. On the contrary, higher drug dosages with shorter exposure periods would also be beneficial when performing downstream assays involving protein and gene expression, as these assays are necessary to investigate drug mechanisms, and are generally conducted shortly upon drug exposure to capture intracellular molecular changes and events.

Despite the fact that most toxicants may initiate cell damage or cell death, the cellular proteins that are involved in control of cell cycle and apoptosis are the final arbiters of cell fate. This study also demonstrates the ability of ACA to induce cell line-based arresting variations in cell cycle distribution patterns following treatment. The effect of ACA in terms of cell cycle arrest in this study has also been consistent with past reports in myeloma cells, indicating cell cycle arresting effects at the G_0_/G_1_ phase [19]. The appearance of a sub-G_1_ apoptotic peak describes a systematic anti-proliferative effect brought upon by ACA, where various cell division activities governed by various CDKs and cyclin proteins are halted, followed by cellular breakdown through the expression of apoptotic effector proteins. Additionally, cell cycle arrest incidences also provide a clear indication that the p53 pathway is somewhat involved, hence a probable target of ACA. This anti-proliferative modulation of ACA is desirable, and provides a more gradual killing of cancer cells in comparison to certain anti-cancer drugs such as cisplatin and gemcitaine-HCl, which in general, imposes a more aggressive effect through the direct interference of DNA replication as means of cellular termination.

The outer membrane protrusion of phosphatidylserine, a common phosphoprotein which is typically inaccessible in viable cells, is detected by annexin V-FITC, and indicates the early stages of apoptosis. This hallmark is part of a sequence of events leading to the loss of membrane integrity, hence allowing fluorescence PI dyes to permeate into the cell to bind nuclei acid, marking the entry of late apoptosis and secondary necrosis [20]. The ability of ACA to induce apoptosis was then confirmed with the conventional DNA fragmentation assay. Unlike necrotic cells where DNA is randomly degraded by a range of nucleases, apoptotic cells go through a systematic breakdown of DNA by endonucleases at specific exposed points of the DNA that are not protected by histone protein complexes [21]. This study showed that DNA fragmentation of cancer cell lines following ACA treatment was consistent with both previous MTT and flow cytometry assays, where partial killing of cells were observed within 24 hrs of exposure.

## 3. Experimental

### 3.1. General

All spectral data were obtained on the following instruments: IR on a Perkin Elmer RX1 FT-IR spectrometer, UV on a Shimadzu UV-160A UV-Visible Recording Spectrophotometer, NMR on a JEOL (Japan Electronic Optics Laboratory Co. Ltd., Tokyo, Japan) JNM-LA400 FT-NMR spectrometer system (400 MHz) and MS on a Shimadzu GC-MS spectrometer (HP 6890 Series Mass Selective Detector and HP 6890 Series GC System).

### 3.2. Chemicals

RPMI-1640, fetal bovine serum (FBS) and all antibiotics were purchased from Lonza Inc. (USA). MTT (3-(4,5-dimethyl-2-thiazolyl)-2,5-diphenyl-2H-tetrazolium bromide) reagent, Annexin V-FITC/PI apoptosis detection kit, propidium iodide (PI), RNase A and Suicide Track™ DNA Ladder Isolation Kit were purchased from EMD Chemicals Inc. (Calbiochem, San Diego, CA, USA). 

### 3.3. Plant Materials

The rhizomes of wild *Alpinia conchigera* Griff were collected from Jeli, province of Kelantan, east-coast of Peninsular Malaysia. The sample was identified by Halijah Ibrahim from the Institute of Biological Science, Faculty of Science, University of Malaya. A voucher specimen (KL5049) was deposited in the Herbarium of Chemistry Department, Faculty of Science, University of Malaya.

### 3.4. Extraction and Isolation

Air-dried and powdered rhizomes of *Alpinia conchigera* (2.1 kg) were extracted with dichloromethane (7.0 L) at room temperature (72 hrs). The solvent was evaporated *in vacuo* to give the dichloromethane extract, which was subjected to column chromatography (CC) on silica gel (Merck Kiesegel 60) eluting with a stepwise gradient of hexane-ethyl acetate (100:0 → 50:50). The fractions were collected separately and concentrated *in vacuo* at 40 °C. The fractions with similar TLC profiles were pooled together to give six sub-fractions which were then subjected to further chromatographic analysis, which yielded 1’-(*S*)-1’-acetoxychavicol acetate as the major constituent. The structure of this compound was determined based on comparison of its spectral data with those reported in the literatures [22,23,24,25,26]. 

### 3.5. 1’-(S)-1’-Acetoxychavicol Acetate:

A yellowish oil, Calculated for C_13_H_14_O_4_, 234.2479; Found 234, 192, 150, 149, 132, 104, 77. IR (CCl_4_) υ_max_, cm^-1^: 1761, 1645, 1234. UV λ_max_, nm: 304.5. ^1^H-NMR (CDCl_3_), δ: 2.08 (3H, *s*), 2.27 (3H, *s*), 5.22 (2H, *dd*, *J* = 10.0 Hz), 5.98 (1H, *m*), 6.23 (1H, *d*, *J* = 5.8 Hz), 7.03 (2H, *d*, *J* = 8.8 Hz), 7.33 (2H, *d*, *J* = 8.8 Hz). ^13^C-NMR (CDCl_3_), δ: 21.2 (CH_3_), 21.3 (CH_3_), 75.6 (CH), 117.2 (CH_2_), 121.7 (2CH), 128.5 (2CH), 136.1 (CH), 136.5 (C), 150.5 (C), 169.4 (C), 169.7 (C).

### 3.6. LCMS Analysis

The LCMS analysis was done using Shimadzu LCMS-IT-TOF instrument (Columbia, MD, USA) equipped with a binary pump, an automatic injector and a photodiode array detector (SPD-M20A). The separation was carried out on a Waters Xbridge C18 column (50 × 2.1 mm, 2.5 μm). A binary gradient solvent system of double-distilled water (eluent A)—acetonitrile (eluent B) was used as follows: 100% A and 0% B (0.0 min), 0% A and 100% B (6.0 min), 100% A and 0% B (8.5–10.0 min). A flow-rate of 0.5 mL/min was used and absorbance was detected at 254 nm. The solution was filtered through Whatman 13 mm, 0.2 µm nylon membrane syringe filters before use. 

### 3.7. Cultivation of Cells

A total of five tumour cell lines were used in this study. Human breast adenocarcinoma (MCF-7) and oral squamous carcinoma (HSC-2 and HSC-4) were obtained from the Cancer Research Initiative Foundation (CARIF, Malaysia), while human hepatocyte carcinoma (HepG2) and epidermoid cervical carcinoma (CaSki) cells were obtained from the University Malaya Medical Centre (UMMC, Malaysia). Human mammary epithelial cells (HMEC) (Lonza, USA) were used as normal cell controls. All cells were cultured in DMEM except MCF-7 cells which were cultured as monolayers in RPMI 1640, supplemented with 10.0% (v/v) FBS, 100.0 U/mL penicillin and 100.0 µg/mL streptomycin. Cultures were maintained in a humidified CO_2_ incubator at 37 °C in 5.0% CO_2_ and 95.0% air. 

### 3.8. Cell Viability Assay

Cell viability was determined using the MTT assay which measures mitochondrial activity in viable cells ACA was dissolved in DMSO to a final concentration of 10.0 mM. Briefly, 2.0 × 10^4^ cells were treated in triplicates on 96-well plates in the presence or absence of ACA at final concentrations of 10.0 µM to 80.0 µM up to 30 hrs. Final DMSO concentration in each experiment was maintained below 0.05% (v/v) to prevent solvent induced cytotoxicty. 20.0 μl of MTT dye reagent (5.0 mg/ml) was added to each well and cells were incubated in the dark at 37 °C. After 2 hrs of incubation, the media containing excess dye was aspirated and 200.0 μL of DMSO was added to dissolve the purple formazan precipitates. A microtiter plate reader (Tecan Sunrise^®^, Switzerland) was used to detect absorbance at a test wavelength of 570 nm, with a reference wavelength of 650 nm. 

### 3.9. Cell Cycle Analysis

Cell cycle analysis was performed using PI based staining methods. Briefly, ACA-treated and untreated cells were fixed in ice-cold 70.0% (v/v) ethanol and kept at –30 °C. Staining of nuclear DNA content was conducted by adding PI (50.0 μg/mL) and RNase A (10.0 mg/mL), followed by incubation at 37 °C for 30 mins in the dark. Fluorescence from a population of 1.0 × 10^4^ cells was detected using the BD FACSCalibur™ flow cytometer at 488 nm and examined on the CellQuest Pro (IVD) software (Becton Dickenson, Mountain View, CA, USA).

### 3.10. Annexin-V Apoptosis Assays

Detection on the various stages of apoptosis was conducted using the Annexin V-FITC/PI apoptosis detection kit according to manufacturer’s protocol. Briefly, media binding reagent containing FITC-conjugated annexin V anti-coagulant (200.0 μg/mg) was added to ACA treated and untreated cells, followed by 15 mins incubation at room temperature. All samples were centrifuged and re-suspended in 1X cold binding buffer and PI (30.0 μg/mL). Detection of signals from a 1.0 × 10^4^ cell population was obtained using the BD FACSCalibur™ flow cytometer and examined on the CellQuest Pro IVD software. 

### 3.11. DNA Fragmentation Assays

Total DNA was extracted from both untreated and treated cells using the Suicide Track™ DNA Ladder Isolation Kit according to the manufacturer’s protocol. Extracted DNA was analyzed on a 1.0% (w/v) agarose gel electrophoresis and stained with ethidium bromide. Fragmentation of DNA was observed under UV illumination and visualized using a gel documentation system (Alpha Inotech, USA).

### 3.12. Statistical Analysis

Results were expressed as mean values with ± standard error of the mean (SEM). All data were performed in triplicates and analyzed using one-way ANOVA, where differences were considered significant at *p* ≤ 0.05.

## 4. Conclusions

This study describes the initial drug development process chain beginning from compound isolation to biological pre-screening assays, and finally to apoptotic assays, confirming the apoptotic inducing effects of ACA on various cancer cell lines, the identification of pathways and genes modulated are vital in the drug development process chain in order to fully understand its apoptotic-inducing machinery. The knowledge and understanding on how ACA potentiates apoptosis as shown in this study is of great importance in further understanding the mechanisms underlying tumourigenesis. This knowledge will provide the basis for newly targeted therapies, hence giving cancer researchers a better insight for future chemotherapeutic approaches.
